# Yellow Fever Vaccine–Associated Viscerotropic Disease: A Unique Case of Atraumatic Splenic Rupture

**DOI:** 10.1155/crdi/7286972

**Published:** 2025-10-13

**Authors:** Eadbert Nortey, Edmund M. Bediako, Geraldine K. Mould, Abigail Mills-Annoh, Kwame Ekremet, Priscilla Kyei-Baffour, Ama Ekem, Jemima C. A. Clarke, Eugene F. E. K. Apaloo, Susan Quartey-Papafio

**Affiliations:** Emergency Department, University of Ghana Medical Centre, Accra, Ghana

**Keywords:** abdominal pain, splenic rupture, vaccine, viscerotropic disease, yellow fever

## Abstract

Atraumatic splenic rupture is an uncommon, life-threatening abdominal emergency involving the rupture of the spleen in the absence of obvious trauma. This case report provides a summary of a 48-year-old male who presented to the Emergency Department of the University of Ghana Medical Centre with syncope and hemodynamic instability one week after receiving a yellow fever vaccine. A diagnosis of an atraumatic splenic rupture was eventually confirmed by imaging, and a splenectomy was done. This report aims to provide valuable insights into the recognition and management of atraumatic splenic rupture, a potential adverse event associated with the yellow-fever vaccine. It also highlights the importance of prompt diagnosis and timely intervention in such life-threatening cases.

## 1. Introduction

Splenic rupture typically results from trauma; however, there have been very few reported cases of rupture occurring in the absence of prior trauma [1]. It may occur in a pathologic spleen associated with certain disease entities, commonly hematologic malignancies, viral infections, and local or systemic inflammatory conditions, or in a normal spleen with no underlying pathology. Atraumatic or spontaneous splenic rupture is a relatively rare clinical entity that poses a potentially life-threatening situation due to its tendency to go unnoticed [2]. Furthermore, splenic rupture as a presentation of viscerotropic disease (VTD), an adverse event associated with yellow fever vaccine, is much less common. This case report documents a rare presentation of VTD leading to atraumatic splenic rupture in a 48-year-old man following yellow fever vaccination.

## 2. Case Presentation

A 48-year-old male presented to the Emergency Department via ambulance following two syncopal episodes immediately after disembarking a long-distance international flight. Prior to these symptoms, he had a one-week history of intermittent fever, general malaise, intermittent dry cough, and mild dyspnea, which he noticed a few hours after receiving a yellow fever vaccine. He had no prior history of trauma and no significant past medical history or family history of VTD.

On initial examination, the patient appeared acutely ill, moderately pale, jaundiced, fully conscious, but notably lethargic with increased work of breathing. He had a respiratory rate of 30 cycles per minute with an oxygen saturation of 88% on room air. His pulse rate was 87 beats per minute, and his blood pressure was 98/54 mmHg. Chest examination revealed reduced air entry in the lower zones of both lungs. Abdominal assessment showed mild distension and severe tenderness in the left hypochondriac region. A bedside ultrasound scan demonstrated minimal fluid in the rectovesical pouch.

Initial management included resuscitation with crystalloids, vasopressors, high-flow oxygen, intravenous antibiotics, and analgesia. A series of investigations followed, including a comprehensive panel of laboratory investigations, a CT pulmonary angiogram, and a contrast-enhanced abdominopelvic CT scan.

**Figure 1:**
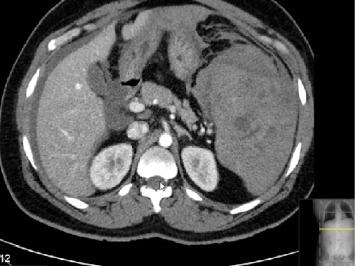
Contrast-enhanced abdominopelvic CT scan showing splenic hematoma with capsular breach.

Initial laboratory investigations were notable for severe anemia (Hb 6 g/dL), leukocytosis (27.77 10∗3/μL), mild thrombocytopenia (119 10∗3/μL), creatinine (1.45 mg/dL), total bilirubin (1.42 mg/dL), INR 1.7, prothrombin time 18.5 s, and markedly elevated D-dimers. The CT pulmonary angiogram ruled out a pulmonary embolism and any masses or pathologies in the chest. The contrast-enhanced abdominopelvic CT (Figure 1) revealed an enlarged spleen with a peri-splenic hematoma, splenic capsular breach, and free intraperitoneal fluid. A diagnosis of atraumatic splenic rupture was therefore made. Yellow fever RNA test was done after 7 days from vaccination and was negative. Other investigations like retro screen were negative.

**Figure 2:**
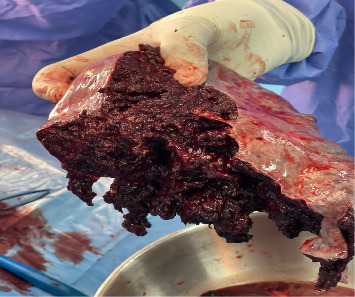
Photo of the ruptured spleen taken during exploratory laparotomy.

The patient's condition acutely deteriorated, resulting in a cardiac arrest. Resuscitation was performed according to ACLS protocol, with return of spontaneous circulation after 20 min. He was transfused with two units of blood and underwent an emergency laparotomy and splenectomy to remove the ruptured spleen (Figure 2), after which he was admitted to the intensive care unit (ICU). However, his condition continued to deteriorate, and he died on Day 29 of admission.

## 3. Discussion

The yellow fever vaccine is a live-attenuated vaccine that contains a weakened form of the virus and is recommended by the WHO for people living in endemic areas. This vaccine has been associated with VTD, with an estimated incidence of about 0.3 cases per 100,000 doses distributed [3]. VTD is defined as acute multiple organ system dysfunction that occurs after vaccination. It has been exclusively described with yellow fever vaccination and as such has been referred to as yellow fever–associated viscerotropic disease (YEL-AVD). It has an incubation period of 1 to 8 days after vaccination [4]. The clinical presentation includes high-grade fever, nausea, vomiting, malaise, arthralgia, diarrhea, and dyspnea [5]. Laboratory investigation may show thrombocytopenia, elevated hepatic enzymes, elevated bilirubin, and increased creatinine levels. In the late phase, patients develop disseminated intravascular coagulation (DIC), cardiovascular instability, and renal and/or respiratory failure [5]. The precise mechanism of VTD is unknown [5]. Host factors that increase the risk of developing VTD include advanced age, thymus disease such as thymoma and myasthenia gravis, and female sex.

Based on WHO recommendations, the Brighton Collaboration provides diagnostic and causality criteria for VTD, defining three levels of diagnostic certainty. This case presentation meets the Brighton Classification criteria for Level 1, with three major criteria: oxygen saturation ≤ 88% in room air, use of vasopressors to maintain systolic blood pressure, and an INR ≥ 1.5. Other minor criteria met include jaundice and increased respiratory rate.

The yellow fever RNA is detectable in the serum during the first 3 to 4 days after vaccinations, with levels subsequently declining [4]. However, when overt symptoms appear, viral RNA is typically undetectable. An IgM antibody response triggered by the vaccine can be detected from Day 4 to 7 days postvaccination, often peaking by Day 14 [4]. Antibodies are present in nearly 90% of individuals by Day 10 and in nearly all individuals by Day 30 [6]. In the presented case, yellow fever RNA testing was done 7 days postvaccination, at which time the patient had overt symptoms, and the result was negative. In this report, RNA testing was limited to blood samples and was not performed on spleen tissue, which may reduce sensitivity for detecting tissue-specific viral replication [7, 8]. However, this does not undermine the diagnostic utility of blood RNA testing, as both sample types provide complementary information and have established roles in the assessment of VTD [9–11].

Additional viral testing such as EBV, CMV, or Mycoplasma, was not performed which would have expanded our scope of evaluation. However, this patient did not have any remote or recent history of note. Furthermore, detailed travel history was not available, with the only documented recent travel being to the United States, limiting the ability to evaluate other potential exposures.

Atraumatic splenic rupture, defined as the rupture of the spleen in the absence of obvious trauma, was first documented in medical literature in the 19th century [12]. The incidence is less than 4% and has been reported more frequently in males than in females [13]. The associated mortality rate ranges from 12.2% to 20% [14]. Numerous theories have been proposed to explain the mechanisms behind atraumatic splenic rupture. Some include the presence of an underlying disease at the time of rupture, long-standing silent passive congestion leading to occult rupture, or rapid splenic arterial dissection into splenic parenchyma due to degenerative changes. Other mechanisms include a sudden increase in intra-abdominal pressure—caused by coughing, exercise, or vomiting that may generate enough force to rupture the spleen [14]. Atraumatic splenic rupture may possibly arise from a combination of these mechanisms [2]. It is most probable that the patient experienced DIC, as evidenced by thrombocytopenia, prolonged prothrombin, and elevated D-dimer levels. This likely resulted in the generation and deposition of fibrin in microvasculature, causing microvascular thrombi in the spleen, which led to severe splenic congestion and eventual rupture. Additionally, his cough may have contributed to a sudden increase in intra-abdominal pressure, further precipitating the splenic rupture.

Splenic rupture presents nonspecifically, typically with left upper quadrant abdominal tenderness, with or without distension, a rapid drop in blood pressure, and syncope. In the absence of antecedent trauma, the diagnosis of splenic rupture may be missed. In the case described above, the patient presented with left upper quadrant pain along with syncopal episodes, hypotension, and hemodynamic instability, but splenic rupture was not considered due to the absence of antecedent trauma. The diagnosis is often missed because atraumatic splenic rupture can mimic other abdominal pathologies [2]. Failure to maintain a high index of suspicion may result in a delayed diagnosis, which can be potentially fatal [13]. The use of imaging modalities such as ultrasound and abdominal CT scan is crucial for diagnosis, with abdominal CT scans being generally more sensitive and useful in assessing and grading the lesion [1].

Management of splenic rupture, whether traumatic or atraumatic, follows similar management principles. Initial management should focus on stabilizing the patient, which may require aggressive fluid and blood product administration to manage hemodynamic instability. Prompt surgical management, involving emergency laparotomy with splenectomy, is required as definitive management for atraumatic splenic rupture with hemodynamic instability [15].

## 4. Conclusion

The yellow fever vaccine is generally considered a safe vaccine and has reduced the incidence of yellow fever outbreaks in endemic regions since the establishment of mass vaccination campaigns by agencies such as the WHO and UNICEF. Although rare, YEL-AVD is a well-described complication of yellow fever vaccination with potentially life-threatening outcomes. Atraumatic splenic rupture could be a critical presentation of YEL-AVD, and a high index of suspicion should be developed by physicians to be able to promptly detect such rare presentations to improve patient outcomes. Further documentation of passive and active surveillance of vaccination adverse events is needed in endemic regions to build robust pharmacovigilance databases. A limitation of our study is the lack of information on the vaccine manufacturer and the absence of yellow fever antibody titers, which may restrict the interpretation of vaccine-related outcomes. Nevertheless, official documents from the Food and Drugs Authority of Ghana indicate that Sanofi Pasteur is the supplier of the yellow fever vaccine [16].

## Data Availability

Data sharing is not applicable to this article as no new data were created or analyzed in this study.
